# Cell Interplay in Osteoarthritis

**DOI:** 10.3389/fcell.2021.720477

**Published:** 2021-08-03

**Authors:** Zihao Li, Ziyu Huang, Lunhao Bai

**Affiliations:** ^1^Department of Orthopedics, Shengjing Hospital of China Medical University, Shenyang, China; ^2^Foreign Languages College, Shanghai Normal University, Shanghai, China

**Keywords:** cartilage, subchondral bone, synovium, infrapatellar fat pad, stem cell, exosome, osteoarthritis

## Abstract

Osteoarthritis (OA) is a common chronic disease and a significant health concern that needs to be urgently solved. OA affects the cartilage and entire joint tissues, including the subchondral bone, synovium, and infrapatellar fat pads. The physiological and pathological changes in these tissues affect the occurrence and development of OA. Understanding complex crosstalk among different joint tissues and their roles in OA initiation and progression is critical in elucidating the pathogenic mechanism of OA. In this review, we begin with an overview of the role of chondrocytes, synovial cells (synovial fibroblasts and macrophages), mast cells, osteoblasts, osteoclasts, various stem cells, and engineered cells (induced pluripotent stem cells) in OA pathogenesis. Then, we discuss the various mechanisms by which these cells communicate, including paracrine signaling, local microenvironment, co-culture, extracellular vesicles (exosomes), and cell tissue engineering. We particularly focus on the therapeutic potential and clinical applications of stem cell-derived extracellular vesicles, which serve as modulators of cell-to-cell communication, in the field of regenerative medicine, such as cartilage repair. Finally, the challenges and limitations related to exosome-based treatment for OA are discussed. This article provides a comprehensive summary of key cells that might be targets of future therapies for OA.

## Introduction

Osteoarthritis (OA) is a common degenerative disease of the joints that causes chronic pain and motor dysfunction and affects the quality of life of more than 300 million people worldwide (GBD 2017 Risk Factor Collaborators, 2018). OA also poses a considerable economic burden on patients and is a major public health problem. OA’s current treatment strategies include non-drug treatment (e.g., exercise, weight reduction, and physiotherapy), drug treatment, and surgery (Bannuru et al., 2019; Kloppenburg and Berenbaum, 2020). Non-pharmacological therapies are used for patients in the early stages of OA to delay its development. However, the effects of these approaches on early symptoms are limited, particularly on structural diseases (Cutolo et al., 2015). On the other hand, medications, including pain relievers and non-steroidal anti-inflammatory drugs, are prescribed to control the pain, preserve functional capacity, and improve the quality of daily life. However, because patients with OA are prone to complications, inappropriate drug treatment and multi-drug therapy can increase the risk of side effects (Gore et al., 2011). Meanwhile, surgical treatment is considered for patients with advanced OA; however, this modality is associated with high failure rates and complications, and additional costs. Because the molecular mechanisms underlying OA initiation and progression remain poorly understood, there are no current interventions with satisfactory curative effects that can delay disease progression (Dieppe et al., 2011; Bannuru et al., 2019). Therefore, new insights into the mechanism of OA pathogenesis are required to promote the development of new therapies that meet future clinical needs.

Osteoarthritis was previously considered to be caused by mechanical damage or the habitual overuse of a joint that is an inevitable part of aging. However, it has become increasingly clear that OA is much more complex than a wear-and-tear disease, and various factors such as inflammation, metabolism, and biochemical machinery play an important role in its pathogenesis (Brandt et al., 2006; Martel-Pelletier et al., 2016). Furthermore, aging, obesity, joint damage, and high-intensity activities have been identified as risk factors leading to OA development (Zhang and Jordan, 2008; Silverwood et al., 2015; Martel-Pelletier et al., 2016). Hence, OA is now viewed as a multifactorial disease that involves local and systemic factors and has multiple pathogenetic mechanisms. Therefore, these factors must be considered when exploring new treatment methods for OA.

In addition, rather than merely involving the destruction of articular cartilage, OA is now more accurately thought of as a disease of the whole joint and is characterized by the partial loss of cartilage, thickening of the synovial sac, subchondral bone sclerosis and osteophyte formation, and changes in the structure of joints, ligaments, and surrounding muscles (Pereira et al., 2011; Hunter and Bierma-Zeinstra, 2019). During its development, different tissues within the joint and their interactions contribute to the pathology and clinical symptoms of OA (Burr, 1998; de Lange-Brokaar et al., 2012). Recently, the role of the subchondral bone, which refers to the cortical bone layer under the articular cartilage and the trabecular bone in the lower part of the joint, in OA pathogenesis has attracted increasing attention. Studies have shown that the subchondral bone may affect cartilage degeneration through changes in mechanical stress or paracrine-mediated interaction between the bone and cartilage (Sharma et al., 2013; Zhen et al., 2013; Lin et al., 2019). In an inflammatory environment, synovial fibroblasts (SFB) may affect the formation of osteophytes and the degradation of the cartilage matrix by releasing bone regulatory factors (including BMP-2) and pro-inflammatory factors (such as IL-1β) (Mathiessen and Conaghan, 2017). Infrapatellar fat pad (IPFP) and synovium can also release various pro-inflammatory mediators during inflammation. These mediators not only result in the abnormal structure and function of synovial tissue but also aggravate cartilage damage and the development of OA (Clockaerts et al., 2010; Mathiessen and Conaghan, 2017; Kuang et al., 2020). Therefore, new insights into the interaction and communication among the different cells in the joint may lead to a greater understanding of the disease mechanism of OA and provide new perspectives for the development of OA treatment strategies.

In this review, we first introduce the cells found in joint tissues and their role in OA pathogenesis. Then, we discuss the various ways by which these cells communicate, including paracrine signaling, local microenvironment, co-culture, extracellular vesicles (EVs), and exosomes. Finally, we summarize the recent studies on the therapeutic potential and clinical applications of stem cell-derived EVs for OA treatment. This article provides a comprehensive summary of key cells that might be targets of future therapies for OA.

## Joint Cells and Tissues

### Cartilage and Chondrocytes

Articular cartilage is the connective tissue located on the surface of the synovial joint and plays a role in lubrication and weight-bearing during joint activities. Due to the lack of a vascular system and limited oxygen and nutrient supply, articular cartilage has low regenerative potential (Sophia Fox et al., 2009; Chen et al., 2017; Singh et al., 2019). In addition to the chondrocytes, which were long considered to be the only cell type within articular cartilage, cartilage stem/progenitor cells (CSPC) have been recently identified in OA cartilage (Williams et al., 2010), representing approximately 10% of the total cells (Alsalameh et al., 2004; Bosserhoff et al., 2014; Riegger et al., 2018). Moreover, single-cell RNA-seq analysis revealed various chondrocyte populations in advanced OA, including homeostatic chondrocytes, proliferative chondrocytes, effector chondrocytes, regulatory chondrocytes, pre-hypertrophic chondrocytes, hypertrophic chondrocytes, and fibrocartilage chondrocytes (Ji et al., 2019).

As the resident cell type in tissues, chondrocytes can form an extracellular matrix mainly composed of aggrecan and type II collagen. However, chondrocytes only account for 1–5% of the total volume of cartilage tissue (Bhosale and Richardson, 2008). Owing to the limited number of cells and their pyknotic nature, damage caused by various risk factors, such as abnormal mechanical load, trauma, and inflammation, can lead to changes in the structure and function of cartilage. Joint degenerative diseases are prone to occur without timely and adequate treatment (Palazzo et al., 2016; Vina and Kwoh, 2018; Li et al., 2019; Singh et al., 2019). Although the composition of articular cartilage is simple, its horizontal-layered structure containing chondrocytes with various morphologies and different distribution and secretion characteristics remains well-organized.

Changes in the metabolic state of chondrocytes lead to the imbalance of collagen synthesis and degradation, cartilage degeneration, chondrosenescence, and an intra-articular inflammatory environment, ultimately leading to OA (Martin and Buckwalter, 2002). The metabolic changes in chondrocytes may exhibit distinct characteristics and have been described in four clinical OA phenotypes. First, the inflammation-associated OA phenotype is characterized by a low degree of inflammation (Scanzello, 2017). The continuous accumulation of pro-inflammatory mediators leads to the excessive production of reactive oxygen species and mtDNA damage and drives the catabolic reaction in the chondrocytes, subsequently disrupting the balance between cartilage repair and damage. The second phenotype is the mechanical overload-associated OA phenotype. Normal physiological load is important to maintain chondrocyte function and ECM metabolic balance. Mechanical overloading is thus harmful to chondrocytes and results in a weakened anabolic response, decreased extracellular matrix (ECM) synthesis, and enhanced catabolic response, thereby stimulating the synthesis of matrix metalloproteinases (MMPs) (Loeser et al., 2012). Metabolic syndrome-associated OA phenotype is characterized by increased fasting plasma glucose concentration, increased triglyceride level, decreased high-density lipoprotein level, and/or hypertension (Dickson et al., 2019). The occurrence and development of OA can be positively affected by increasing the production of fat-derived pro-inflammatory mediators, such as advanced glycation end-products (Oren et al., 2011). The last is the aging-associated OA phenotype. The catabolism of aging chondrocytes is active, and disruption of the interplay between autophagy and the inflammasome is observed in an inflammatory environment (Salminen et al., 2012). In addition, the decline in chondrocyte mitochondrial function is accompanied by decreased chondrocyte autophagy and increased apoptosis (Blanco et al., 2018). The proliferation and synthesis ability of chondrocytes in aging articular cartilage also decreases; however, their ability to produce pro-inflammatory mediators and MMPs remains unchanged (Loeser, 2009). Overall, the initiation and progression of OA are closely related to the phenotypic changes in chondrocytes.

### Synovium and Synovial Cells

Increasing evidence has shown that the mutual communication between different tissues in the joint is essential for maintaining joint homeostasis. The communication between synovium and cartilage not only contributes to OA symptoms but is also a key factor in disease pathogenesis. The morphology and cell composition of the synovium are often used as biomarkers for the development of OA. Although the synovial tissue may not be affected in the early-stage of OA, many patients with advanced OA suffer from severe synovitis (Wenham and Conaghan, 2010).

In healthy joints, the synovium is mainly composed of two types of synovial cells. Type A macrophage-like synovial cells, which are relatively small, mainly have a phagocytic function and produce pro-inflammatory cytokines. Meanwhile, type B fibroblast-like synovial cells provide structure, nutrition, and lubrication and represent approximately 75% of the cells in the synovium (Firestein and McInnes, 2017). In addition, fibroblasts-like synovial cells can migrate to the site of tissue remodeling and interact with ECM molecules via specific surface receptors (Pap and Bertrand, 2012). They can perceive and respond to the changes in the composition and structure of the surrounding synovial tissue by adjusting their interaction with and the production of ECM components. In the initiation of OA, the intimal lining layer becomes hypertrophic and is infiltrated by macrophages, fibroblasts, mast cells, T cells, B cells, dendritic cells, and neutrophils, leading to a 5- to 10-fold increase in cell density (Culemann et al., 2019). These infiltrating cells promote the production of pro-inflammatory cytokines and catabolites, thereby changing the composition of synovial fluid, an important source of these pro-inflammatory mediators in OA (Falconer et al., 2018). In the synovial fluid of OA patients, the proportion of macrophages is relatively low, whereas that of mast cells is relatively high (de Lange-Brokaar et al., 2012; Robinson et al., 2016; Xie et al., 2019).

#### Macrophages

Synovitis can occur at both the early and late stages of OA (Sellam and Berenbaum, 2010) and is characterized by the accumulation of macrophages in the intimal lining layer (Sun A. R. et al., 2016). Macrophages are heterogeneous and plastic immune cells that produce chemokines and cytokines in inflamed joints. Macrophages can be activated by various stimuli, including pro-inflammatory (IL-1β, TNF-α, and IL-6) and immunomodulatory cytokines (IL-4 and IL-10) (Wang et al., 2014; Dutta et al., 2020) and abnormal mechanical forces, which are mostly produced during stress or cell damage (Liu et al., 2018). Activated macrophages are generally classified into two distinct phenotypes, namely, classically activated/inflammatory (M1) type and alternatively activated/immunomodulatory (M2) type.

Polarized M1 macrophages can generate a large amount of pro-inflammatory cytokines, nitric oxide (NO), and reactive oxygen species, thereby enhancing host defense response (Mosser, 2003; Genin et al., 2015). However, excessive activation of M1 macrophages can lead to autoimmune diseases and tissue damage (Mosser and Edwards, 2008). M2 macrophages are mainly present in the subsiding phase of inflammation and are responsible for producing anti-inflammatory cytokines and eliminating apoptotic cells. In addition, M2 macrophages can produce osteogenic growth factors, such as bone morphogenetic protein 2 (BMP-2; an effective promoter of osteogenic differentiation of MSCs belonging to a subclass of the TGF-β family), (Champagne et al., 2002; Li C. J. et al., 2018) TGF-β, (Assoian et al., 1987) 1,25-dihydroxy vitamin D3, (Kreutz et al., 1993) and osteopontin (Takahashi et al., 2004).

Therefore, the imbalance in the ratio between these two kinds of cells in OA may be related to the initiation and progression of OA (Xue et al., 2019). The significantly increased number of M1 macrophages in synovial tissues (Sun et al., 2017) and the high proportion of M2 macrophages have clinical diagnostic significance for OA (Chen et al., 2020). However, it is difficult to assess the polarization state of synovial macrophages before OA occurs or even in its early stages. In late-stage OA, M1- and M2-like macrophages can coexist in the joint synovium and adjacent adipose tissues. However, the role of macrophages in OA initiation and progression is unquestionable. Macrophages play an important role in the occurrence of OA through inflammatory factors, cytokines, and proteins, whether it is inflammatory or mechanical injury (Bondeson et al., 2010).

#### Mast Cells

Mast cells (MCs), a type of immune cells that reside in tissues, play a pivotal role in allergic reactions (Galli and Tsai, 2012). The synovial fluid of patients with OA showed an increased number of MCs and increased concentration of certain MC mediators, such as histamine and tryptase (Bridges et al., 1991) (Buckley et al., 1997). The role of MCs in bone metabolism remains controversial (Urist and McLean, 1957). In MC-deficient mouse models, MCs were involved in the occurrence and development of OA (Schubert et al., 2015; Kroner et al., 2017) and fracture healing and may also be involved in regulating the production of osteoclasts (Behrends et al., 2014; Kroner et al., 2017). Many MC mediators can regulate or induce bone metabolism by inhibiting osteoblast activity (such as IL-1, TNF) and/or promoting osteoclastogenesis (such as histamine, TNF, IL-6) (Biosse-Duplan et al., 2009; Pietschmann et al., 2016). MCs can also play a role in maintaining bone homeostasis. For instance, the transforming growth factor-β (TGF-β) can stimulate the production of osteoblasts, while IL-12 and interferon-γ (IFN-γ) can inhibit the formation of osteoclasts (Pietschmann et al., 2016).

Several clinical studies have reported the increased expression of genes involved in MC differentiation and activity in the synovial tissues of OA patients (Wang et al., 2019). MC-deficient mice were protected from inflammation and cartilage destruction of OA; however, MC implantation in engraftment reversed this protection (Wang et al., 2019). In addition, the inhibition of tryptase activity in wild-type mice reduced the concentration of pro-inflammatory mediators, such as IL-6, IL-1β, IL-8, and MMP-3. Furthermore, the synovial MCs of patients with OA can secrete TNF-α following stimulation with the high-affinity receptor of IgG (Lee et al., 2013). In a cross-sectional cohort study, H1 antihistamine treatment was associated with decreased prevalence of OA (Shirinsky and Shirinsky, 2018). These results confirm the critical role of MC in the occurrence and development of OA and indicate that MCs may be a potential therapeutic target for OA.

### Subchondral Bone and Osteoblasts and Osteoclasts

The degeneration and degradation of articular cartilage had long been considered the leading cause of OA, and many treatment strategies have been developed to protect the cartilage. Although the relationship between cartilage degeneration and subchondral bone destruction is close (Thielen et al., 2019), not all patients with OA exhibit abnormalities in the articular cartilage bone. In addition, in the aging OA phenotype, the imbalance in chondrocyte metabolism occurs before abnormal subchondral bone remodeling (Peffers et al., 2020). In contrast, in the trauma-induced OA phenotype, the early micro-injury in subchondral bone is detected first (Barton et al., 2017).

However, increasing evidence demonstrates that maintaining the integrity and remodeling balance of the articular subchondral bone can combat cartilage degeneration to restore homeostasis in joint tissues (Castañeda et al., 2012; Hoshi et al., 2017). Thus, exploring the mechanism underlying subchondral bone remodeling in OA can provide new insights for developing treatments for early-stage OA. The microstructural changes in articular subchondral bone in OA include the formation of subchondral bone cysts, bone marrow edema-like lesions, and osteophytes caused by early bone loss, late bone sclerosis, and histopathological changes (Li et al., 2013). These changes are caused by chondrocytes, osteoblasts, osteoclasts, endothelial cells, and the subchondral bone microenvironment (Henrotin et al., 2012).

Osteoblasts differentiate from mesenchymal cells and undergo four stages of maturation, namely, preosteoblasts, osteoblasts, bone-lining cells, and bone cells (Clarke, 2008; Katsimbri, 2017). The phenotype and activity of OA osteoblasts in subchondral bone are altered. For example, the levels of OCN, RANKL, (Kwan Tat et al., 2008) insulin-like growth factor 1, (Hilal et al., 1998) transforming growth factor β1, (Abed et al., 2017) vascular endothelial growth factor (Corrado et al., 2013), and alkaline phosphatase activity are elevated in OA, which subsequently lead to osteoclastogenesis, sclerosis (Wang et al., 2013) and angiogenesis. Osteoclasts are multinucleated cells derived from bone marrow myeloid progenitor cells and are mainly responsible for bone resorption and formation (Teitelbaum, 2000; Katsimbri, 2017). During osteoclast formation, progenitor cells are recruited to specific parts of the bone surface to differentiate into osteoclasts (monocytes) and fuse, thereby forming multinucleated mature osteoclasts. Mature osteoclasts adhere to the old bone area and release hydrogen ions and catalytic enzymes to dissolve the bone.

### Mesenchymal Stem Cells

Mesenchymal stem cells (MSCs) are multipotent progenitor cells that originate from the mesoderm. MSCs by default can’t regenerate bona fide articular hyaline cartilage (it’s primarily fibrous cartilage and hypertrophy) (Buhrmann et al., 2010). However, MSCs can contribute to cartilage and bone repair, and their function in immune regulation and organ regeneration has been extensively studied (Levy et al., 2020). MSCs can be roughly divided into three types: embryonic stem cells, pluripotent stem cells, and adult stem cells (Vizoso et al., 2017).

Pluripotent stem cells are found in bone-related tissues, such as the bone marrow, synovium, infrapatellar fat pad, and adipose tissues (Pers et al., 2015). Bone marrow-derived MSCs (BMSCs) are the most well-characterized pluripotent stem cells. As early as 2002, stem cell therapy based on the *in vitro* expansion of autologous BMSCs has been used to treat OA. Although there was no significant difference in clinical results, improvement in symptoms was observed from arthroscopic and histological findings (Wakitani et al., 2002). *In vitro*, synovial MSCs (SMSCs) exhibit a particularly high capacity for cartilage differentiation (Shirasawa et al., 2006; Kurth et al., 2007). Studies have shown that SMSCs from OA patients can repair cartilage through allogenic tissue-engineered constructs in both *in vitro* and *in vivo* models. In experimental animal models, the injection of SMSCs into the joint cavity achieved a similar effect (Kondo et al., 2019; Enomoto et al., 2020; Dragoo et al., 2012). Infrapatellar fat pad (IPFP), a column of fat tissue located behind the patella, and synovium are involved in the occurrence and development of intra-articular diseases, such as OA (Favero et al., 2017). As the MSCs/stromal cells derived from IPFP (Kouroupis et al., 2019) are similar to SMSCs, (To et al., 2019) IPFP MSCs are speculated to have the ability for tissue repair (Galipeau et al., 2016), indicating IPFP as a potential target for joint diseases (Attur et al., 2010). Its potential in chondrogenic, osteogenic, and adipogenic lineages has been reported (Sun et al., 2018). *In vitro*, the chondrogenic differentiation ability of IPFP MSCs was greater than that of BMSCs, adipose tissue MSC (AMSCs), and UC-MSCs (Ding et al., 2015). AMSCs can promote cartilage regeneration and regulate inflammation. Because they are versatile and readily available, AMSCs are an excellent source of cells for OA treatment (Kim and Koh, 2018; Lee et al., 2019). However, the mechanism by which AMSCs induce cartilage regeneration remains unclear. Current evidence indicates that AMSCs regulate the local microenvironment through paracrine nutritional factors, thereby making it more favorable for regeneration and repair and subsequently delaying cartilage degradation and improving joint function (Damia et al., 2018). Previously, a small number of MSC-like progenitor cells-chondrogenic stem cells/progenitor cells (CSPCs) were detected in cartilage tissues (Alsalameh et al., 2004; Fickert et al., 2004; Koelling et al., 2009). Because they share similar properties with BMSCs, CSPCs are speculated to be involved in cartilage regeneration. CSPC migration occurs upon cartilage damage, and their proliferation and immune regulation capabilities are enhanced (Seol et al., 2012; Riegger et al., 2018).

### Induced Pluripotent Stem Cells and Tissue-Engineered Cells

Induced pluripotent stem cells (iPSCs) can provide an unlimited cell source for tissue engineering and are an attractive substitute for primary cells. iPSCs are characterized by a high degree of plasticity and promising differentiation potential and have promising potential in cell therapy. Patient-specific iPSCs can be engineered to minimize the autoimmune response, making them an almost ideal cell source for cell-based therapy. Studies on cartilage tissue engineering of iPSCs have demonstrated their utility for functional cartilage repair and as models for studying cartilage pathology (Diekman et al., 2012; Willard et al., 2014). iPSCs provide a platform for identifying candidate, patient-specific OA therapeutic agents (Lietman, 2016). For example, iPSCs reprogrammed from somatic cells (Takahashi et al., 2007; Yu et al., 2007) could generate endless OA patient-specific stem cells for drug research. Moreover, iPSCs isolated from the tissues of OA patients could differentiate into the cartilage, which provides opportunities for cartilage tissue research (Wei et al., 2012; Lach et al., 2014; Nam et al., 2018). However, there are no clinical studies published about cartilage cell therapy using iPSCs. iPSCs not only have excellent proliferation and differentiation capabilities similar to other stem cells but also do not cause immune rejection and ethical issues (Moradi et al., 2019). Therefore, more studies are required to improve the future applications of iPSC-derived chondrocytes in OA replacement therapy.

## Various Mechanisms of Cellular Crosstalk in OA Pathogenesis

### Microenvironment, Paracrine Signals, and Co-culture Method

#### Cartilage and Subchondral Bone

In joints with OA, some blood vessels of the subchondral bone can penetrate calcified cartilage and even invade non-calcified cartilage (Chen et al., 2015). Osteoclast precursors invade the area of hypertrophic cartilage and interact with its cells to reshape the cartilage matrix and form an ossification center (Tonna et al., 2016). In addition, mature osteoclasts could regulate nearby chondrocytes, which destroys the connection between the bone and cartilage and degrade articular cartilage via cysteine proteases and matrix metalloproteinases (Löfvall et al., 2018). Osteoclasts could aggravate cartilage damage by regulating chondrocytes. The expression level of TGF-β1 in osteoclasts was upregulated in a time-dependent and dose-dependent manner under mechanical stimulation. Upon co-culture with osteoclasts, chondrocytes showed aggravated apoptosis. The injection of TGF-β1R inhibitor into the abdominal cavity of rats with OA effectively reduced chondrocyte apoptosis and cartilage degradation (Zhang R. K. et al., 2018). TGF-β1 was not derived from osteoclastic bone resorption but was transported from the subchondral bone to the cartilage layer via diffusion or blood circulation to adversely affect chondrocytes. The cartilage can also obtain calcium–phosphate complexes from subchondral bone via p38, ERK1/2, nuclear factor- kappa B (NF-κB), signal transducer, and activator of transcription 3 (STAT3) to increase the production of MMP-13 in chondrocytes (Jung et al., 2018).

Chondrocytes can also promote the loss of subchondral bone by regulating osteoclasts. Abnormal mechanical stress could induce IL-1β production in primary chondrocytes (Fujisawa et al., 1999). IL-1β increased the expression of receptor activator of NF-κB ligand (RANKL) through osteoblasts, thereby indirectly inducing the generation and maturation of osteoclasts (Cao et al., 2016). In an OA model induced by destabilization of the medial meniscus (DMM), chondrocytes produced large amounts of TNF-α and IL-6 (Pearson et al., 2017). TNF-α activates NF-κB and c-Jun NH2-terminal protein kinase (JNK) in a RANKL-independent manner to directly induce osteoclast differentiation (Kobayashi et al., 2000) and indirectly induce its production (Tanaka et al., 2005). In an *in vivo* OA model, the expression of high-mobility group box 1 (HMGB1) was detected in chondrocytes (Aulin et al., 2020). As demonstrated by the bone development of *HMGB1^–/–^* hypertrophic chondrocytes in the mouse growth plate, endochondral bone formation was disrupted due to the delayed invasion of osteoclast precursors into the primary ossification center (Taniguchi et al., 2007). In addition, senescent chondrocytes and hypertrophic chondrocytes produced pro-inflammatory mediators, catabolic enzymes, and chemokines, collectively known as the senescence-associated secretory phenotype (SASP), (Rim et al., 2020) to affect subchondral osteoclast lineage cells.

#### Infrapatellar Fat Pad and Synovium

In OA, IPFP and nearby synovium also experience inflammatory infiltration and hyperplasia (Favero et al., 2017). IPFP releases IL-6, IL-8, prostaglandin F2a (PGF2α), and TNFα, which subsequently causes fibrosis of the synovium (Bastiaansen-Jenniskens et al., 2013; Eymard et al., 2014). *In vitro*, fibroblast-like synovial cells of OA patients pre-treated with TGFβ or PGF2α inhibitors were cultured in the conditioned medium derived from IPFP tissues for 4 days and exhibited different migration and proliferation abilities. Hyperplasia and fibrosis also occurred (Bastiaansen-Jenniskens et al., 2013). In addition, adipocytes derived from IPFP could regulate macrophages and CD4 + T cells infiltrating into the synovium by secreting lipids (Ioan-Facsinay et al., 2013; Klein-Wieringa et al., 2013). The free fatty acids found in the conditioned medium derived from IPFP adipocytes improved the proliferation of CD4 + T cells and their ability to produce IFN-γ. These free fatty acids could also reduce the secretion of IL-12p40 cytokine by macrophages (Klein-Wieringa et al., 2013); IL-12p40 is a chemoattractant that can induce macrophage inflammation and fibrosis (Cooper and Khader, 2007).

Macrophages have been used as potential therapeutic targets, and their pharmacological depletion and phenotypic changes in IPFP and synovium have been explored (Fernandes et al., 2020; Wu et al., 2020). Macrophages can be induced to polarize back to another anti-inflammatory M2 phenotype. Although there are no reports on the MSC-mediated direct regulation of the phenotype of synovial macrophages in patients with OA, MSCs have been demonstrated to block the activation of M1 macrophages and promote the polarization of M2 macrophages to inhibit inflammation *in vitro* (Harrell et al., 2019). MSCs first migrate to the tissue injury site and promote the polarization of M2 macrophages by secreting a large number of cytokines, chemokines, and growth factors (Fernandes et al., 2020), thereby enhancing the repair of damaged tissues. The intra-articular injection of MSCs could downregulate the level of iNOS in macrophages and reduce the formation of M1 macrophages (Hamilton et al., 2019). Interestingly, in OA joints, M1 macrophages subsequently inhibited the proliferation and viability of MSCs, enhanced the immune response, and ultimately aggravated cartilage degradation.

Infrapatellar fat pad can also interact with the cartilage. The main limitation of MSC-based cartilage constructs is the induction of a hypertrophic phenotype during *in vivo* differentiation, which leads to endochondral ossification (Scotti et al., 2013; Correa et al., 2015; Feng et al., 2018). However, 8 weeks after implementing hybrid structures in which IPFP MSCs and articular chondrocytes were co-cultured in nude mice, cartilage mineralization was reduced, and the phenotype was stable (Mesallati et al., 2015). IPFP MSCs could accumulate a large amount of sGAG on articular cartilage agarose gels, thereby improving the mechanical properties of tissue-engineered articular cartilage constructs (Mesallati et al., 2017).

#### Cell Co-culture

Co-culture of chondrocytes and synovial cells has been used in cartilage research as an *in vitro* model for OA. Co-cultured chondrocytes and synovial cells stimulated by pro-inflammatory cytokines interact via calcium signaling and paracrine pathway to maintain the homeostasis of chondrocytes. This co-culture method allows for the accurate evaluation of the role of anti-inflammatory or chondroprotective molecules in the articular cartilage (Beekhuizen et al., 2011). The co-culture of osteoblasts and chondrocytes can also be used to study the role of chondroprotection (via delaying the onset of cartilage degradation) in bone remodeling. Paracrine signals are also used to maintain the physiological state and phenotype of the cells (Thysen et al., 2015). MSCs promote specific dedifferentiation due to their pluripotency. When co-cultured with other cells, different cell pathways can be analyzed together with articular chondrocytes with cell secretion markers (Hendriks et al., 2007). However, the co-cultivation method also has certain limitations, including restricted growth of cells and high cost.

### Intercellular Signaling by EVs

Extracellular vesicles (EVs) are nano-sized communication messengers secreted by cells that transmit biological signals between cells and are mainly divided into three categories, namely, exosomes, microvesicles, and apoptotic bodies. EVs have received increasing research attention in the field of regenerative medicine. Increasing studies have demonstrated potential value for alleviating inflammation and promoting tissue repair and regeneration (Bjørge et al., 2017).

Extracellular vesicles are small double lipid membrane vesicles with a diameter of approximately 30–2000 nm that can carry various biologically active molecules, such as RNA subtypes (mRNA, microRNA, and lncRNA), DNA fragments, lipids, proteins, and enzymes (Lamichhane et al., 2015). Most cells, such as those in connective tissues and MSCs, can produce and secrete EVs into various biological fluids, such as the blood and synovial fluid (Rani et al., 2015). Once released, EVs can take effect immediately or be transported to a distant place. EVs communicate with the recipient cell by producing the same effect as their donor cell.

Exosomes are small vesicles with a diameter of approximately 30–150 nm derived from the endosomal compartment. They are the most widely studied type of EVs (Théry et al., 2002). Endosomal membrane invaginates to form multivesicular bodies containing intraluminal vesicles. When multivesicular bodies fuse with the plasma membrane, intraluminal vesicles are secreted as exosomes into the extracellular space. Microvesicles are slightly larger than exosomes, with a diameter ranging between 50 and 1000 nm (De Jong et al., 2014). Because microvesicles are directly shed from the plasma membrane, their markers on the membrane surface are also the same as the donor cells. Similar to exosomes, microvesicles can transport biologically active molecules to recipient cells. Apoptotic bodies are the largest type of EVs, with a diameter of ≥1000 nm, and are formed in the late-stage of apoptosis (De Jong et al., 2014). Apoptotic bodies may function as biomarkers. However, at present, the connection between regenerative medicine and cells remains unclear.

Extracellular vesicles serve as mediators of intercellular communication (Raposo and Stoorvogel, 2013; De Jong et al., 2014). EVs interact with the surface receptors of recipient cells through their transmembrane proteins to activate downstream intracellular signaling pathways. EVs can also be directly endocytosed by recipient cells to release their contents (Jaiswal et al., 2014). The type of donor cells and the environment in which they are located, such as being in a state of stress, can affect EVs’ function and contents (de Jong et al., 2012). The biological functions of EVs and their biogenesis require more studies. Increasing studies in regenerative medicine have focused on the production of protective and pro-regenerative EVs, particularly in cartilage repair.

Studies have revealed the role of EVs in OA pathogenesis, inflammation, and cartilage regeneration and have demonstrated their potential implications for joint disease therapy (Zhang S. et al., 2018). EVs derived from different types of joint cells participate in maintaining joint homeostasis and can initiate and promote the progression of OA (Murphy et al., 2018). Macrophages and leukocytes infiltrating the synovium could interact with fibroblast-like synovial cells through EVs (Malda et al., 2016). The activated fibroblast-like synovial cells then transmit inflammatory signals, such as cytokines and enzymes, to the macrophages and leukocytes, thus forming a feedback loop that further aggravates OA. These EVs could also cause the degradation of the extracellular matrix and result in changes in the subchondral bone. In an *in vitro* OA model, EVs enhanced cartilage anabolism and relieved inflammation (Wang et al., 2017), thereby delaying cartilage degradation and the progression of OA (Zhu et al., 2017). In addition, EVs protected chondrocytes (Headland et al., 2015) and regulated the physiological activities of various types of immune cells (Lo Sicco et al., 2017), indicating their anti-inflammatory effects. The induction of a regenerative immune phenotype and enhanced metabolic level of chondrocytes promote the formation of type II collagen-rich cartilage that can repair cartilage defects in rat and rabbit OA models (Zhang et al., 2016; Zhang S. et al., 2018). Hydrogel encapsulation delivers EVs at more accurate positions and higher doses, thereby significantly enhancing repair ability (Liu et al., 2017).

Recently, the application of stem cell-derived EVs in the treatment of joint damage and OA has received increasing attention. They have been derived from the bone marrow (Cosenza et al., 2018; Vonk et al., 2018), adipose tissue (Lo Sicco et al., 2017), synovium (Zhu et al., 2017), or pluripotent cells, including embryonic stem cells (Zhang et al., 2016; Zhang S. et al., 2018) and iPSCs (Liu et al., 2017; Zhu et al., 2017). Exosomes are the main research target in EVs. The difference between exosomes and microvesicles derived from the same cell has been investigated (Cosenza et al., 2017, 2018). Moreover, EVs containing both exosomes and microvesicles have been analyzed (Headland et al., 2015; Lo Sicco et al., 2017). In addition to sharing similar biological functions with stem cells, stem cell-derived EVs offer significant advantages due to their small size and low immunogenicity. Issues associated with direct cell injection can also be avoided. EVs do not pose the risk of antigen presentation due to differentiation into specific cell types such as MSCs, which allows them to be used in allogeneic therapy. In addition, the biologically active cargoes inside MSC-derived EVs are more stable, and problems such as senescence following expansion or cartilage calcification following induction are eliminated.

### Exosomes Derived From Joint Tissues

Exosomes maintain homeostasis (Gao et al., 2018) and facilitate cell-to-cell communication in diseases (Li Z. et al., 2018). The source and content of exosomes determine their functions and biological characteristics. Exosomes secreted by therapeutic cells help treat diseases, while those released by cells in the pathological microenvironment accelerate the disease process. In this section, we discuss the various kinds of exosomes derived from joint tissues and their biological effects in OA.

#### Cartilage-Derived Exosomes

Osteoarthritis chondrocytes release extracellular articular cartilage matrix vesicles with a diameter of 100 nm and participate in the pathologic mineralization of articular cartilage (Anderson, 2003; Jubeck et al., 2008). Exosomes and articular cartilage matrix vesicles share similar features, including size, morphology, and lipid and protein content (Shapiro et al., 2015), suggesting that they exhibit homologous functions with respect to cell communication (Ni et al., 2019). Exosomes derived from normal primary chondrocytes (D0 exosomes) could restore mitochondrial function and enhance immune infiltration by increasing the ratio of M2/M1 macrophages. The intra-articular injection of D0 exosomes effectively suppressed the occurrence and development of OA (Zheng et al., 2019). OA chondrocyte-derived exosomes stimulated the activation of inflammasomes in macrophages and released mature IL-1β via the miR-449a-5p/ATG4B/autophagy pathway, thereby inducing synovitis and exacerbating OA (Ni et al., 2019). In addition, exosomes derived from chondrocytes could achieve efficient ectopic chondrogenesis of cartilage progenitor cell (CPC) constructs, representing a novel cell-free therapeutic approach for cartilage regeneration (Chen et al., 2018).

#### Synovial-Derived Exosomes

Microvesicles derived from neutrophils have been shown to penetrate cartilage, implying that EVs from the synovium could meditate the crosstalk between the synovium and cartilage (Headland et al., 2015). Kato et al. employed IL-1β to stimulate normal SFB, isolated the secreted exosomes, and co-cultured them with chondrocytes. They found that the expression of catabolic-related genes, such as *MMP13* and *ADAMTS-5*, significantly increased, whereas that of anabolic-related genes, such as *COL2A1* and *ACAN*, significantly decreased. In addition, these exosomes promoted the production of proteoglycan from cartilage explants (Kato et al., 2014). These findings indicate that synovial-derived exosomes could induce OA-like phenotype both *in vivo* and *in vitro*.

Exosomes derived from other synovial cells, such as macrophages, have also been studied. For instance, the effects of salazosulfapyridine and methotrexate on the proteome of exosomes produced by a human synovial sarcoma cell line (SW982) have been investigated. Tsuno et al. observed that these anti-rheumatic drugs altered the protein profiles of SW982-derived exosomes and inhibited the effect of IL-1β on the exosomal proteome (Tsuno et al., 2016).

#### Subchondral Bone-Derived Exosomes

Exosomes derived from cells in the subchondral bone, including osteoblasts, osteoclasts, and bone marrow mesenchymal stem cells, regulate the microenvironment of subchondral bone (Li et al., 2016; Sun W. et al., 2016). Osteoblasts in the subchondral bone of patients with different degrees of OA secreted exosomes positive for HSP70, CD9, and flotillin-1 (exosomal markers) and a diameter ranging between 30–150 nm. In addition, these exosomes contained a large number of miRNAs, such as miR-135a-3p, miR-210-5p, miR-885-3p, and miR-1225-5p. Exosomes derived from cells in other subchondral bones also have diagnostic value.

#### Therapeutic Potential of Stem Cell-Derived Exosomes in OA

Stem cells, such as BMSCs and AMSCs, promote cartilage regeneration and have been used in clinical trials for OA treatment (Lee and Wang, 2017; De Bari and Roelofs, 2018). The safety and feasibility of intra-articular injection of MSC have been confirmed (Di Matteo et al., 2019), which can partially relieve knee joint pain (Yokota et al., 2019) and improve the knee society clinical rating system (KSS) and the outcome score of OA (Jo et al., 2017; Kim et al., 2020). Stem cells exert therapeutic effects mainly through their paracrine functions, such as the secretion of EVs (Phinney and Pittenger, 2017). In this section, we summarize the recent studies on exosomes derived from different types of stem cells, focusing on their roles in the occurrence and development of OA and their therapeutic potential.

##### BMSC-derived exosomes

Exosomes derived from BMSCs (BMSC-Exos) can promote the regeneration and repair of damaged cartilage and subchondral bone (Mianehsaz et al., 2019; Asghar et al., 2020). The exosomes and microvesicles from TGFβ3-pre-treated BMSCs increased the expression of anabolic markers and decreased the levels of catabolic marker genes in osteoarthritic chondrocytes. In addition, these BMSC-derived exosomes could prevent osteoarthritic chondrocytes from undergoing apoptosis (Cosenza et al., 2017). BMSC-Exos could be taken up by chondrocytes to abolish damage to the mitochondrial membrane potential and IL-1β-induced apoptosis (Qi et al., 2019). BMSC-Exos could also affect the phenotype of other cells in OA joints by inhibiting the activity of osteoclasts in subchondral bone and activating macrophages in the synovium (Li et al., 2020), and suppressing the proliferative activity of SFB pre-treated with IL-1β and increasing its apoptosis. In an *in vivo* OA model, the injection of BMSC-Exos into the joint cavity abrogated the damage and degradation of cartilage and subchondral bone and decreased synovial tissue proliferation and inflammatory cell infiltration, thereby alleviating the symptoms of OA (Jin et al., 2020).

Genetic modification or drug intervention can influence the effect of exosomes on recipient cells by regulating the secretion and contents of exosomes (Ma et al., 2017; Liu and Su, 2019). Exosomes secreted by BMSCs overexpressing miR-92a-3p were collected and applied to chondrocytes. The expression levels of SOX9, COL2A1, and aggrecan increased, whereas those of RUNX2 and MMP13 decreased, indicating that modified BMSC-Exos greatly enhance cartilage repair (Mao et al., 2018). In addition, *in vivo*, and *in vitro* experiments showed that kartogenin-pre-treated BMSC-Exos more significantly enhanced cartilage formation and damage repair than normal BMSC-Exos (Liu et al., 2020).

##### BMSC-derived exosomes

*In vitro* synovial mesenchymal stem cells (SMSCs) possess an exceptionally high capacity for cartilage differentiation (Kurth et al., 2007). One study using allogenic tissue-engineered constructs reported that SMSCs from OA patients effectively enhanced cartilage repair (Koizumi et al., 2016). In animal OA models, the injection of SMSC into the joint cavity inhibited the occurrence and development of OA (Ozeki et al., 2016; Kondo et al., 2019). Exosomes derived from SMSC (SMSC-Exos) could not only induce the proliferation and migration of chondrocytes but also reduce the secretion of ECM. Interestingly, SMSC-Exos transfected with miR-140-5p blocked damage to ECM, effectively reduced joint damage, lowered the OARSI score, and delayed the occurrence and development of OA (Tao et al., 2017). In addition to maintaining cartilage homeostasis, SMSC-Exos can regulate bone remodeling (including subchondral bone changes and osteophyte formation) by reducing glucocorticoid-induced fat cell accumulation, trabecular bone loss, and bone marrow necrosis. In addition, SMSC-Exos could partially reverse proliferation arrest and glucocorticoid-induced apoptosis of BMSCs (Guo et al., 2016).

##### IPFP MSC-derived exosomes

Infrapatellar fat pad plays a key role in knee joint function and pathology. IPFP-derived MSCs (IPFP MSCs) have been suggested as promising cell sources for OA treatment owing to their potent capability for cartilage regeneration (Buckley et al., 2010; Koh and Choi, 2012). In a DMM-induced OA mouse model, exosomes derived from IPFP MSCs effectively reduced cartilage damage and improved abnormal gait. RNA sequencing analysis of the exosomes revealed high miR-100-5p levels, indicating that exosomal IPFP MSCs may inhibit the mTOR pathway via miR-100-5p to regulate chondrocyte phenotype (Wu et al., 2019). The physiological and pathological effects of other exosomes in the IPFP, such as those secreted by adipocytes, also have research value.

##### AMSC-derived exosomes

Although the mechanism by which AMSCs induce cartilage regeneration is unclear, mounting evidence suggests that AMSCs regulate the cartilage microenvironment by secreting paracrine growth factors (Damia et al., 2018). EVs, including exosomes and microvesicles, mainly mediate the paracrine effects of osteoblasts in OA. AMSC-derived exosomes (AMSC-Exos) reduced the accumulation of senescence-associated β-galactosidase and γH2AX foci in osteoblasts pre-treated with IL-1β, decreased the levels of PGE2 and IL-6, increased that of IL-10, and downregulated the mitochondrial membrane potential (Tofiño-Vian et al., 2017). In addition, AMSC-Exos could inhibit the production of pro-inflammatory mediators, such as TNF-α and NO, and suppress the activity of MMP while enhancing that of anti-inflammatory cytokines, such as IL-10 and type II collagen. These findings indicate the anti-inflammatory and chondroprotective effects of AMSC-Exos (Tofiño-Vian et al., 2018). AMSC-derived EVs (86.46 nm in diameter) promoted the proliferation and migration of OA chondrocytes and maintained the metabolic balance of ECM. In monosodium iodoacetate (MIA) rat and DMM mouse models, the injection of AMSC-derived EVs into the joint cavity effectively delayed OA progression and showed protective effects against cartilage degeneration (Woo et al., 2020). ADSCs-Exos could downregulate the expression of pro-inflammatory genes in SFB and increase anti-inflammatory cytokines, promoting the proliferation and cartilage formation of periosteal cells via miR-145 and miR-221 (Zhao C. et al., 2020). Overall, AMSC-Exos has great therapeutic potential for the treatment of OA.

##### Embryonic mesenchymal stem cell (EMSC)-derived exosomes

Embryonic mesenchymal stem cells are another potential candidate for cartilage regeneration and OA treatment (Mamidi et al., 2016; Gibson et al., 2017). Recently, exosomes derived from embryonic MSCs (EMSC-Exos) have been reported to regulate the phenotype of chondrocytes and delayed OA progression (Zhang S. et al., 2018; Zhang et al., 2019). After successfully isolating and identifying EMSC-Exos, Zhang et al. (2016) injected them into osteochondral defects in rats. After 6 weeks, cartilage and subchondral bone damage were largely reversed, and complete recovery was achieved at 12 weeks. Similarly, exosomes derived from the E1-MYC 16.3 human embryonic stem cell line reduced the production of M1 macrophages and pro-inflammatory cytokines and increased the infiltration of M2 macrophages. In addition, this study observed that EMSC-Exos could be endocytosed by chondrocytes to regulate their chondrocyte proliferation, migration, and matrix synthesis (Zhang S. et al., 2018). In an OA model of the temporomandibular joint in rats, EMSC-Exos reduced inflammation, alleviated early pain, and promoted cartilage repair and subchondral bone healing. The activation of AKT, ERK, and AMPK pathways can also reverse IL-1β-induced production of MMP13 and NO and inhibit sGAG synthesis (Zhang et al., 2019). TGF-β1 increases miR-135b levels in EMSC-Exos, thereby reducing the expression of Sp1 to promote the proliferation of chondrocytes and accelerate cartilage repair (Wang et al., 2018).

##### Exosomes derived from other stem cells

Exosomes derived from amniotic fluid stem cells (AFSC-Exos) in an MIA-induced OA model improved the pain tolerance, induced the restoration of regular hyaline cartilage, and inhibited the polarization of M1 macrophages, suggesting that AFSC-Exos can regulate inflammation (Zavatti et al., 2020). Meanwhile, those derived from umbilical mesenchymal stem cells (UMSC-Exos) induced chondroprotective effects, including increased proliferation and migration of chondrocytes, increased ECM synthesis, and reduced cell apoptosis. UMSC-Exos produced from 3D culture enhanced cartilage repair compared to those from 2D culture (Yan and Wu, 2020). Exosomes secreted by MSCs derived from pluripotent stem cells (with a diameter of approximately 50–150 nm; iMSC-Exos) significantly promoted the proliferation and migration of chondrocytes and had improved efficacy for OA treatment compared with SMSC-Exos (Zhu et al., 2017).

However, how the exosomes in joint tissues and cells participate in OA initiation remains unclear. In addition to their positive therapeutic effects, exosomes in the OA microenvironment may exert unwanted effects, including promotion of inflammation and inhibition of cartilage repair. Therefore, it is also imperative to explore the mechanism of “negative” exosomes in OA. A recent study on exosomes in the plasma and synovial fluid reported their diagnostic value in patients with OA (Zhao and Xu, 2018). However, the exosomes shared by these two tissues could not distinguish between early-stage and late-stage OA. Notably, the expression level of exosomal lncRNA PCGEM1 in synovial fluid was significantly higher in patients with advanced OA than in those with early OA and was higher in early OA, indicating that exosomal lncRNA PCGEM1 from synovial fluid may be a powerful indicator for distinguishing early-stage and late-stage OA. lncRNA PCGEM1 acts as sponge lncRNA targeting miR-770 and promotes the proliferation of synovial cells (Kang et al., 2016). In general, the clinical utility of exosomes as diagnostic biomarkers for OA diagnosis is in its preliminary stages.

### Cell Tissue Engineering

Recent attempts to differentiate iPSCs derived from OA patients into chondrocytes have been conducted. Generally, the addition of growth factors, such as TGF-β, FGF-2, BMP, and WNT3A, and paracrine factors, such as Ihh and Runx, to the culture medium is necessary to drive iPSCs to the chondrogenic lineage (Yamashita et al., 2018; Zhao Y. et al., 2020). Currently, four main methods are available: (1) transformation of iPSCs into MSC-like cells and their differentiation into chondrocytes (Nejadnik et al., 2015); (2) co-culture of MSCs derived from iPSCs with primary chondrocytes (Qu et al., 2013); (3) formation of embryoid bodies (EBs) (Nakagawa et al., 2009); (4) cultivation of iPSCs in a medium that mimics the physiological environment during development (Diekman et al., 2012). Studies aiming to form iPSCs through EB and co-culture them with chondrocytes have been conducted (Wei et al., 2012). First, the chondrocytes of OA patients were reprogrammed into OA-iPSCs by lentivirus induction. After the formation of EB, they were continually cultured in the chondrogenic medium for 14 days. Subsequently, the iPSCs were transfected with a lentivirus carrying TGF-1β and inoculated on alginate matrix-coated dishes. After culturing for another 14 days, TGF-1β/iPSCs were subcutaneously injected into the back of mice. Ectopic cartilage tissue was observed at 6 weeks after transplantation.

Engineered cartilage tissue from chondrocytes, when transiently transfected circuits activate the *PTGS2* gene, immunomodulatory IL-4 is produced, thereby representing a new immunomodulatory method (Nims et al., 2021). Autologous articular chondrocytes are an established cell-based tissue engineering strategy for treating knee cartilage or osteochondral defects (Brittberg et al., 1994). Most of the current scaffolds or biomaterials contain MSCs that can undergo chondrogenic differentiation and are used as clinically relevant chondrogenic implants to repair cartilage defects. However, after their implantation in the body, differentiated chondrocytes showed a hypertrophic phenotype (collagen X, MMP13) and induced ectopic bone formation (Pelttari et al., 2006). Therefore, the production of articular chondrocytes with stable, extracellular matrix and phenotype is the main goal of *in vitro* cartilage tissue engineering.

Macrophages are among the main types of cells that affect joint homeostasis and have been applied for the development of related cell engineering technologies. In addition to using biomaterial scaffolds to regulate macrophage-induced inflammation, macrophages themselves have been utilized for drug delivery or treatment. The intrinsic homing ability of macrophages allows their migration to the site of inflammation or injury in OA. Using this feature, autologous M1 macrophages are used to deliver nanoparticle-encapsulated drugs to induce transient phagosome maturation arrest (Visser et al., 2019). The regular use of clustered interspaced short palindromic repeats (CRISPR)-Cas9 genome editing to create a cell-autonomous system (”SMART” cell), which is derived from mouse induced pluripotent stem cells (miPSCs), has also been attempted. Chondrocytes can automatically regulate inflammation in both *in vivo* and *in vitro* OA models. When “SMART” chondrocytes receive specific targeting signals (such as inflammatory cytokines IL-1 or TNF-α), they released corresponding biological drugs, such as IL-1Ra and or soluble TNFR1, to relieve inflammation (Brunger et al., 2017; Pferdehirt et al., 2019). In the same way, the design of self-regulating “SMART” macrophages enables these cells to not only automatically home to the inflammation/injury site but also to have cytokine-activated feedback-controlled capabilities. Thus, targeted drugs can be effectively delivered to treat joint diseases (Adkar et al., 2017). Overall, cell tissue engineering is a powerful tool to develop new modalities for OA treatment.

## Conclusion and Prospects

**FIGURE 1:**
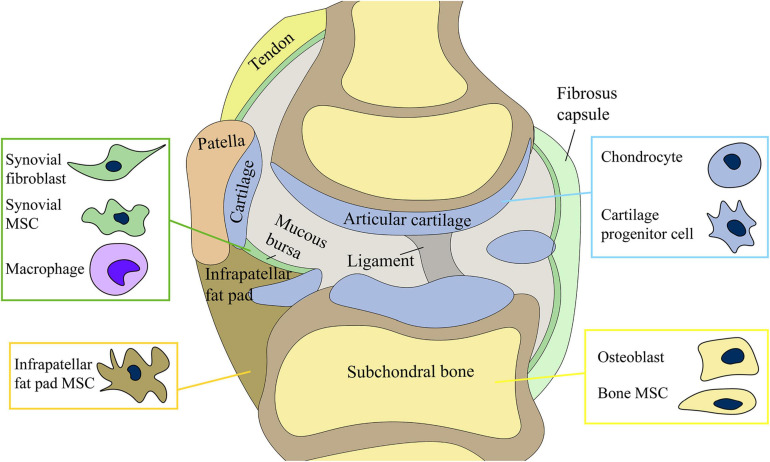
Joint cells) include chondrocytes, osteoblasts of subchondral bone, synovial mesenchymal stem cells (MSCs) and fibroblasts, and infrapatellar fat pad MSCs. The production and release of exosomes by stem cells may be involved in the regulation of joint homeostasis.

**FIGURE 2:**
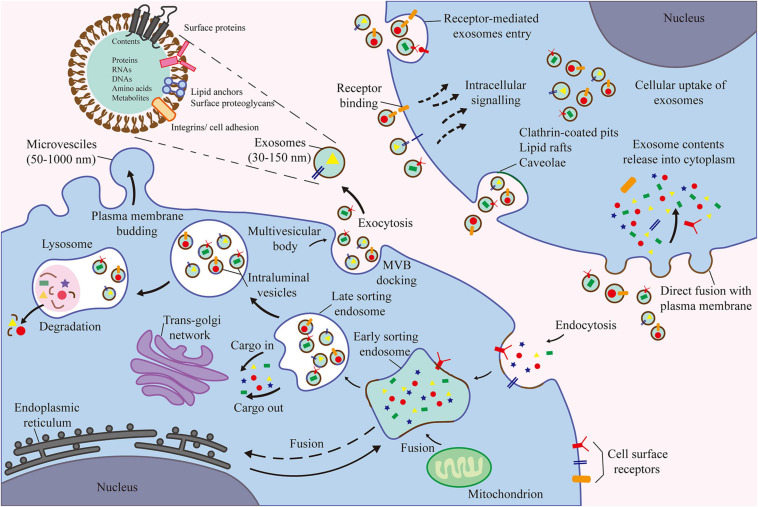
The contents of exosomes are proteins, nucleic acids, amino acids, and metabolites. Extracellular components enter the cell through endocytosis and plasma membrane invagination. Plasma membrane buds are formed on the cavity side and fuse with the components of the endoplasmic reticulum, trans-Golgi network, and mitochondria to form early sorting endosomes. Then, the late sorting endosomes modify the cargo and produce and form various intraluminal vesicles and multivesicular bodies (MVB). Among them, some MVBs degrade after fusion with lysosomes. Other MVB can be transported to the plasma membrane to release intraluminal vesicles as exosomes outside the cell through exocytosis.

This review discusses the functions of different types of cells in the joints and their roles in OA, the interaction among various joint cells and tissues, and the latest cell tissue engineering techniques. Our article provides a comprehensive summary of the complex mechanisms underlying the occurrence and development of OA and potential targets of future therapies for OA (Figure 1). Among the various cell types, MSCs and their secreted products (EVs) are the focus of future research (Figure 2).

Increasing studies have demonstrated the clinical applications of MSCs (Mendicino et al., 2014), such as their role in promoting cartilage repair and delaying the progression of OA (Lee and Wang, 2017). In phase I and II clinical trials, the injection of MSCs into the joint cavity is reportedly reliable and safe. A 5-year follow-up survey demonstrated the efficacy of MSCs in improving cartilage quality and joint function (Yubo et al., 2017; McIntyre et al., 2018). However, the use of MSCs for OA treatment has certain limitations. For example, cell survival and long-term cell behavior after injection are difficult to predict, and the maintenance of cell banks is also a major challenge (Heldring et al., 2015). The quality of MSCs from different donors varies, particularly from elderly or deceased donors who have reduced proliferation capacity and physiological function. In addition, the *in vitro* expansion of MSCs causes senescence, proliferation decline, and even dedifferentiation (Siddappa et al., 2007). MSCs are also “environmentally responsive,” and changes in the microenvironment can cause drastic changes in cell behavior (Murphy et al., 2013). For example, the stimulation of AMSCs by TNF changed the phenotype and resulted in the secretion of pro-inflammatory proteins, which aggravated the inflammatory response (Lee et al., 2010).

Immunotherapy and nanotechnology can help overcome these limitations. At present, genetic engineering techniques, including the use of viral vectors and CRISPR-Cas9 genome editing (Gerace et al., 2017; Pawitan et al., 2020), have been proposed to improve the immune regulation of MSCs. The biological scaffold carrying MSCs expressed IL-1Ra under the influence of exogenous doxycycline, which induces inflammation resistance, contributing to the recovery of degenerative articular cartilage, indicating that cell engineering combined with biological materials can enhance the immunomodulatory ability of MSCs (Glass et al., 2014).

Exploring the function of the products secreted by MSCs, such as EVs, is an alternate direction for OA treatment. The application of stem cell-derived EVs in OA treatment is an emerging field in regenerative medicine. The cargoes delivered by EVs are the same as those of donor cells; however, the former is simpler, more practical, and safer than direct cell transplantation. Exosomes serve as an important mediator of cellular interaction during OA development and have tremendous therapeutic potential. However, this research area has some challenges. At present, there is no direct evidence for the delivery and transfer of endogenous exosomes between cells, which makes identifying the recipient cells of exosomes difficult. The mechanism of exosome production and release in joints also remains unclear, limiting the development of cell therapies that target exosomes. Because cartilage destruction is not severe in early-stage OA, exosomes cannot effectively penetrate the cartilage matrix to interact with cartilage cells. Therefore, current studies on MSC-Exos cell engineering mainly focus on surface chondrocytes, cartilage matrix, synovial cells, and other joint cells that can easily communicate with exosomes.

Future studies on EVs should explore their cell sources, optimized conditions for the production of EVs, their content and biodistribution in the joints, the type of recipient cells or tissues, and their therapeutic mechanisms in OA. Engineered products based on EVs are expected to further promote the interaction between cells, and the intersection between biology and engineering technology can further optimize the function and production of EVs. Finally, it can represent a relatively complete treatment strategy to reduce the burden of OA patients.

A better understanding of the various interactions among cells and tissues in the joint in OA pathogenesis paves the development of future cell-based therapies for OA treatment.

## Author Contributions

ZL conducted the literature review, drafted the manuscript, and prepared the figures. ZH and LB edited and revised the manuscript. All authors have substantially contributed to the article and approved the submitted version.

## Conflict of Interest

The authors declare that the research was conducted in the absence of any commercial or financial relationships that could be construed as a potential conflict of interest.

## Publisher’s Note

All claims expressed in this article are solely those of the authors and do not necessarily represent those of their affiliated organizations, or those of the publisher, the editors and the reviewers. Any product that may be evaluated in this article, or claim that may be made by its manufacturer, is not guaranteed or endorsed by the publisher.
